# A Split Ring Resonator Dielectric Probe for Near-Field Dielectric Imaging

**DOI:** 10.1038/s41598-017-02176-3

**Published:** 2017-05-17

**Authors:** Dmitry Isakov, Chris J. Stevens, Flynn Castles, Patrick S. Grant

**Affiliations:** 1 University of Oxford, Department of Materials, Parks Road, Oxford, OX1 3PH UK; 2 University of Oxford, Department of Engineering Science, Parks Road, Oxford, OX1 3PJ UK

## Abstract

A single split-ring resonator (SRR) probe for 2D surface mapping and imaging of relative dielectric permittivity for the characterisation of composite materials has been developed. The imaging principle, the analysis and the sensitivity of the SRR surface dielectric probe data is described. The surface dielectric properties of composite materials in the frequency range 1–3 GHz have been measured based on the magnetic resonance frequency of the transmission loss of the SRR dielectric probe when in contact with the surface. The SRR probe performance was analysed analytically and using full-wave simulation, and predictions showed close agreement with experiment for composite materials with spatially varying dielectric permittivity manufactured by 3D printing. The spatial and permittivity resolution of the SRR dielectric probe were controlled by the geometrical parameters of the SRR which provided flexibility to tune the SRR probe. The best accuracy of the dielectric permittivity measurements was within 5%.

## Introduction

Composite dielectric materials with temperature stable relative dielectric permittivity, low dielectric loss, high thermal conductivity, and good mechanical properties are of technological interest for applications in microelectronic packaging, electromagnetic shielding, waveguides, antenna and other communication elements^1–3^. Recent progress in manufacturing techniques, including additive manufacturing and 3D printing, allows the fabrication of composite dielectric materials for novel electromagnetic applications with spatially-varying dielectric and/or magnetic properties^4–6^. These intentionally heterogeneous composite materials allow for unusual manipulation of microwaves such as flat lenses, highly directional antennas, cloaks, etc. However, where such heterogeneous functionality is required in the material and component design, there is also a need to verify the as-manufactured spatial distribution of electromagnetic materials properties in order to ensure conformance with the design, and to help the interpretation of the arising electromagnetic behaviour of the final device. Usually the control of the local dielectric properties of the composite material is achieved during the sequential or layer-by-layer manufacture of the component by controlling the local weight or volume fraction of a high relative permittivity within the composite material. Thus, a method for assessing the local electromagnetic properties in the functional composites must provide a spatial and dielectric permittivity resolution that is consistent with the intended design and the resolution of the manufacturing process.

The spatial distribution of the effective dielectric permittivity of composite materials is investigated most commonly using 2D dielectric broadband probe techniques operating at radio and microwave frequencies, based on an open-ended coaxial line reflection method^7, 8^. For example, this method is widely applied for *in vivo* measurements of permittivity in biological tissues for medical imaging and diagnostics^9, 10^. The complex permittivity of the material is extracted from the reflection coefficient using an equivalent circuit of an open-ended coaxial line of the probe in contact with the flat surface of the sample. The typical resolution of this method is related to the physical dimensions of the probe, and typically lies in the range from a few millimetres to a few centimetres.

Microscopy-based techniques have been developed for the mapping of surface dielectric properties with high spatial resolution, typically in the micrometer to sub-micrometer range^11^. The basic principle of these near-field scanning microwave microscopy techniques relates to the perturbation of the resonance of a cavity coupled to a probe tip. The probe is a sharpened metal tip mounted in the centre conductor of a coaxial resonator that extends beyond an aperture formed in the end wall of the resonator^12–14^. The dielectric properties of the materials can be deduced from the variation of the cavity resonance. A micro-strip line resonator probe has also been proposed to increase the resolution of the microscope, which allows the use of different substrate dielectric constants and thicknesses, tapering angles, various apertures, and feed line-to-resonator coupling^15^, giving a spatial resolution as fine as 0.4 *μ*m^16^.

Microwave microscopy may also make use of a narrow resonant slot in a rectangular hollow waveguide serving as a near-field source^17, 18^, and has been used for contactless mapping of the resistivity and the dielectric permittivity of surfaces. The spatial resolution of the slot-type microscope is determined by the slot width, and is generally lower than 100 *μ*m. The spectrum-image method has been used for the 2D mapping of dielectric permittivity, and is based on scanning an electron beam across a sample and obtaining the spatially resolved energy-loss spectroscopy spectrum^19, 20^. Using the Kramers-Kronig relationship, the dielectric permittivity over a broad frequency spectrum may then be deduced from the reconstructed scattering spectra.

Surface dielectric permittivity mapping techniques have been developed based on scanning force microscopy (SFM), with very high spatial resolution. In SFM, the amplitude of the second harmonic of electromechanical vibrations of the tip reflects both electrostriction and capacitance of the system and is, therefore dependent on the dielectric constant of the material. For example, the low-frequency dielectric permittivity of a thin film of SiO_2_ was obtained by measuring the capacitance between the scanning tip and the sample using a capacitance sensor^21^. This principle was also used for imaging variations in the dielectric constant in several piezoelectrics and ferroelectrics^22, 23^ and for monitoring dielectric relaxation in a glassy polymer polyvinyl acetate^24^. For measurements at higher frequencies, the SFM was interfaced with a vector network analyser, and used for quantitative measurements of electrical properties in nanoscale samples, by converting the reflection coefficient *S*_11_ into complex impedance data from which the dielectric permittivity at GHz frequencies could be retrieved^25, 26^.

Despite the relative convenience and maturity of microscopy methods, they are generally not easily used for mapping spatially varying dielectric properties over the large areas (often cms × cms) associated with fabrication materials and devices for operation in the MHz and GHz frequency range. On the other hand, open-ended coaxial line reflection techniques are able to scan large areas, but suffer from a relatively low resolution, limited by the probe sensing area, which is typically no better than a few millimetres. A solution to span the critical millimetre to sub-millimetre spatial range has been proposed using a tapered probe design for a coaxial cavity sensor^27^, or using an electromagnetic sensor based on two coupled spiral inductors to measure, for example, barely-visible impact damage in a carbon-fibre reinforced polymer composites^2^. This latter type of sensor, based on the measurement of the transmission coefficient *S*_21_, provided an approximately 0.4 mm step resolution but only a qualitative 2D map of dielectric permittivity. Table 1 summarises the available dielectric surface imaging techniques in terms of their operating frequency, spatial resolution and other features.

**Table 1:** Summary of dielectric surface imaging techniques.

Detecting element	Operational frequency	Spatial resolution	Features	Refs
Open-ended coaxial	0.3–12 GHz	>3 mm	Non-resonant, *S*_11_	7–10
Coaxial probe with sharpened tip	1.2 GHz	0.3–1 *μ*m	Resonant, requires calibration	13, 14
Coaxial probe with sharpened tip	2.3–18 GHz	20–200 nm	Resonant, *S*_11_	25, 26
Coaxial resonator probe	1.5–2.7 GHz	1 *μ*m	Charge redistribution	12
Micro stripline with sharpened tip	1 GHz	0.4 *μ*m	Resonant, *S*_11_	15, 16
Waveguide slit	10 GHz, 80 GHz	100 *μ*m	Resonant, *S*_11_	17, 18
SFM conducting tip	20 kHz	20–100 nm	Capacitance measurement	22–24
Coupled planar spiral inductors	10–500 MHz	0.4 mm	Non-resonant, *S*_21_, qualitative only	2

This paper presents an alternative 2D surface scanning technique for fast, quantitative measurements of dielectric permittivity in the 1–3 GHz frequency range, over areas of many square centimetres while retaining sub-millimetre spatial resolution. The sensor element is based on a single split-ring resonator (SRR), where the variables of the geometry of the split in the ring provide flexibility in tuning the spatial resolution from 0.1 to 2 mm. We show qualitative and quantitative 2D imaging of the surface electromagnetic properties of large area heterogeneous composite materials produced by 3D printing and other techniques. A further novelty of the SRR probe approach is that, due to the out-of-plane orientation of the excitation electric field across the slit in the resonating ring, the probe is sensitive to anisotropy in the dielectric permittivity and thus can be also used to characterise birefringence of the materials.

## Results and Discussion

### The SRR probe

**Figure 1:**
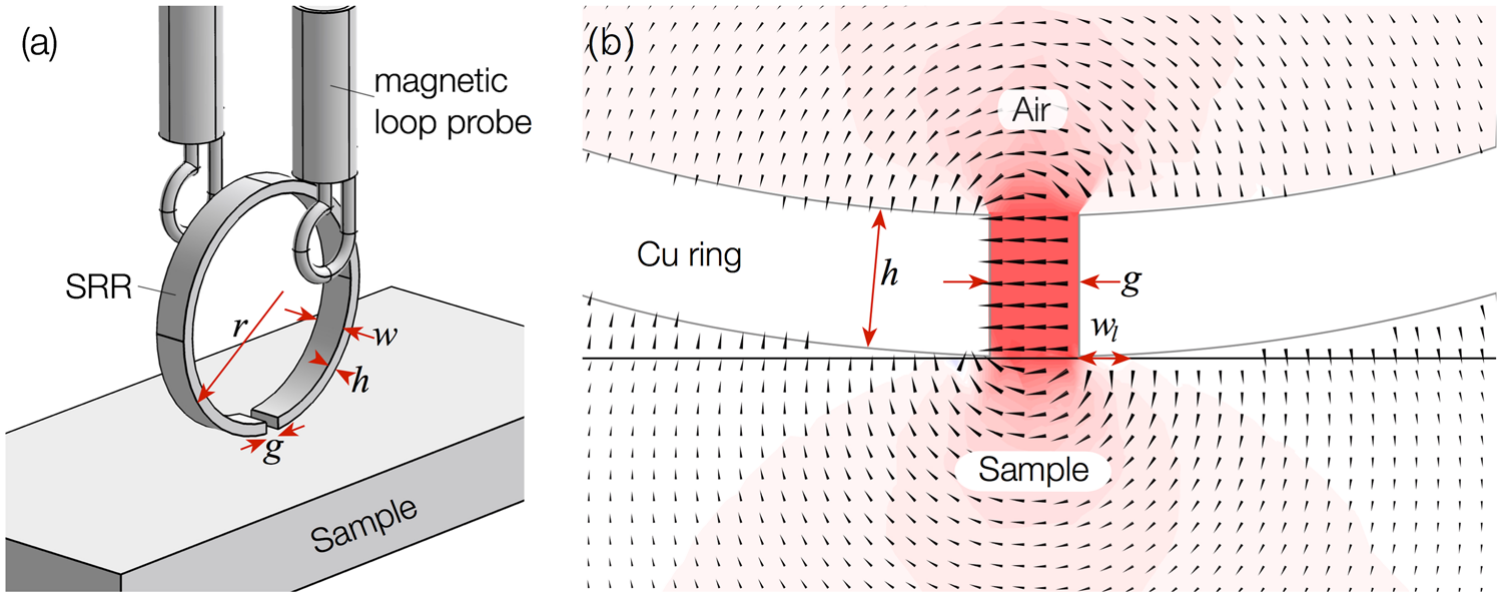
(**a**) Schematic of the experimental 3D arrangement of the SRR probe coupled between two magnetic loop probes and in touching contact with the surface of a material; and (**b**) the zoomed-in cross-section of the SRR in contact with a material with *ε*_*r*_ = 2 − *j* · 0.02. The vectors and color contours represent the direction and strength of the induced electric field in the plane of the cross-section at the resonant frequency, according to full wave simulation.

The SRR consisted of a Cu ring with outer radius *r*, width *w*, height *h*, with a gap width *g* (Fig. 1a). The probing of the surface dielectric permittivity using the SRR is based on the shift of the resonant frequency *f*_0_ of the transmission signal *S*_21_ passing from coupled transmitting and receiving magnetic loops, connected to a vector network analyser (VNA), in between which the SRR is mounted, as the SRR approaches and then rests with minimal load, just touching the surface.

Considering first the case of a SRR in free space, the SRR response is excited by a time-varying magnetic field parallel to the split ring axis, generated by the loop probe connected to VNA ports, and results from a resonant exchange of energy between the electrostatic fields in the capacitive gap and the inductive currents inside the split ring^28, 29^. Thus, the resonator system can be considered as a *LC* circuit composed of an effective inductance *L* and a capacitance *C* with magnetic resonance frequency:

1 $${f}_{0}={\mathrm{(2}\pi \sqrt{LC})}^{-1}.$$

When the ring is then brought into touching contact with a surface, the total capacitance *C* of the SRR arrangement is the sum of the gap capacitance *C*_gap_, the capacitance of the ring surface *C*_ring_, and the capacitance due to the material in tangential contact with the split ring *C*_sample_, so that:

2 $$C={C}_{{\rm{gap}}}+{C}_{{\rm{ring}}}+{C}_{{\rm{sample}}}.$$

Thus, materials with a different dielectric permittivity to air will cause a shift resonant frequency of the SRR. The magnetic inductance *L* and surface ring capacitance *C*_ring_ of the SRR can be calculated according to ref. 30:

3 $$L={\mu }_{0}R(\mathrm{log}\,\frac{8R}{h+w}-\frac{1}{2}),$$

and

4 $${C}_{{\rm{ring}}}=2{\varepsilon }_{0}\frac{h+w}{\pi }\,\mathrm{log}\,\frac{4(r-h)}{g},$$

where *μ*_0_ and *ε*_0_ are the free-space permeability and permittivity and *R* = *r* − *h*/2 is the mean ring radius. The gap capacitance *C*_gap_ is:

5 $${C}_{{\rm{gap}}}={\varepsilon }_{0}\frac{hw}{g}+{\varepsilon }_{0}(h+w+g),$$

where the first part of the right side of equation (5) is a parallel plate capacitance due to the air in the gap, and the second part is a correction due to fringing (*C*_f_) of the electric field at the ring edges^31^.

If the SRR is in contact with a surface (Fig. 1b) with relative dielectric permittivity *ε*_*r*_, the sample capacitance *C*_sample_ can be obtained using a conformal transformation in analogy with co-planar strip lines^32–34^:

6 $${C}_{{\rm{sample}}}={\varepsilon }_{0}\frac{l}{2}[\frac{K({k}_{0}^{^{\prime} })}{K({k}_{0})}+{\varepsilon }_{r}\frac{K({k}_{1}^{^{\prime} })}{K({k}_{1})}],$$

where *l* is the length of the plates of the parallel plate capacitor with relative dielectric permittivity *ε*_*r*_, and *K*(*k*_0_), $K({k}_{0}^{^{\prime} })$, *K*(*k*_1_) and $K({k}_{1}^{^{\prime} })$ are the complete elliptic integrals of the first kind with $k^{\prime} =\sqrt{1-{k}^{2}}$. *k*_0_ and *k*_1_ are defined correspondingly as:

7 $${k}_{0}=\frac{g}{2{w}_{l}+g},$$

8 $${k}_{1}=\frac{\tanh (\pi g/4S)}{\tanh (\pi (2{w}_{l}+g)/4S)},$$

where *S* is the thickness of the dielectric substrate slab, and *w*_*l*_ is the length of the SRR that is in contact with the dielectric sample surface (see Fig. 1b), which can be approximated as:

9 $${w}_{l}=\sqrt{{r}^{2}-{(\frac{h}{32})}^{2}}-\frac{g}{2}.$$

By combining Equations (1) to (9) an analytical expression for *f*_0_ as a function of *ε*_*r*_ and other variables can be obtained, which can be expressed as:

10 $${\varepsilon }_{r}(f)=A+B{f}_{0}^{-2},$$

where coefficients *A* and *B* characterise the geometry of the SRR.

### Numerical and experimental verification

**Figure 2:**
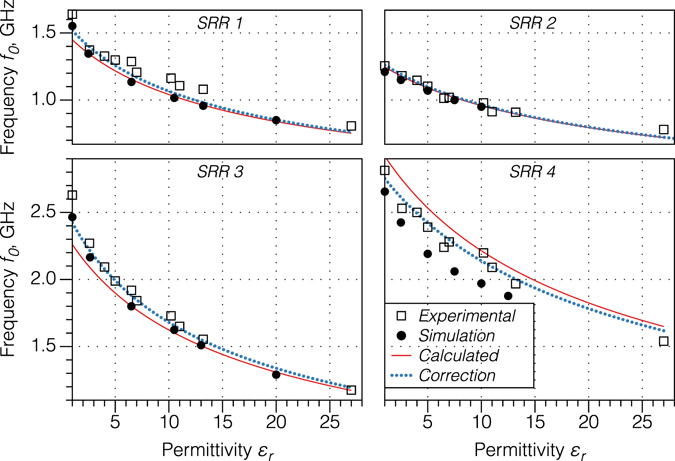
The analytical (red solid curve), measured (open squares) and simulated (filled circles) resonant frequency *f*_0_ of the SRR probe as a function of the dielectric permittivity *ε*_*r*_ of materials for the different SRR geometrical parameters given in Table 2. Corrected curves (blue dotted line) for the analytical approach were obtained by using alternative fringing gap capacitance. The range of dielectric permittivities was provided by using ABS-based polymeric composites with different fractions of micron-scale inorganic powders such as BaTiO_3_, and were previously characterized using a standard split post resonator technique.

Figure 2 shows the dependence of the resonant frequency *f*_0_(*ε*_*r*_) of the SRR probe as a function of the relative dielectric permittivity of a material in contact with the ring for four different geometry SRRs, calculated analytically from Equations (1)–(10). The geometrical parameters of each of the SRR probes are presented in Table 2. Figure 2 also shows both the measured and simulated results for *f*_0_(*ε*_*r*_) obtained for the same ring geometries. The experimental relative permittivities were obtained for the sets of different samples made of polymer-ceramic composites with different weight ratios to provide variations in the overall effective dielectric permittivity. The relative permittivity of each composite was independently obtained in bulk form using a split-post dielectric resonator operating at 15 GHz.

**Table 2:** Geometrical parameters of the SRR probes used in the experiments and simulations shown in Fig. 2.

	Radius *r* [mm]	Width *w* [mm]	Height [mm] *h*	Gap *g* [mm]
SRR 1	10.85	5.01	0.80	0.30
SRR 2	11	2.95	0.90	0.10
SRR 3	7.15	5.02	0.61	0.40
SRR 4	7.35	1.1	0.75	0.25

The analytical calculations of *f*_0_(*ε*_*r*_) were within 5% of experiment for SRRs 2 to 4. The numerically simulated resonant frequencies (filled circles in Fig. 2) were in close agreement with the analytical calculations for all the SRRs investigated. The largest discrepancy between experiment and prediction was 15.8% for SRR 1. The main experimental errors in the technique arise from the accuracy to which the geometry of the SRR may be measured and the flatness of the sample surface (and thus the area of contact). The particular reason why SRR 1 showed relatively poor experimental agreement with theory was not clear, but likely arose because of variable or imperfect contact of the SRR with the surface. Future implementations could use a load control mechanism to ensure consistent touching contact of the SRR with the surface.

Although the polymer-ceramic composites used in this work have a relatively small dispersion of dielectric permittivity in the 1–20 GHz frequency range^5^, deviation of the experimental data from theory may arise due to different measurement frequency. Recall that the reference dielectric permittivities (abscissa axis in Fig. 2) were obtained at 15 GHz, whereas the analysis and the measurements were performed in the vicinity of the lower resonant frequencies, in the range of 1–2.5 GHz. Although the Q-factor (*Q* = *f*_0_/Δ*f*, where Δ*f* is the −3 dB bandwidth of the *S*_21_ response) of the *S*_21_ resonance was in the range of 30–100 (depending on the particular SRR and material), uncertainties in resonant frequency were relatively small (estimated at ±0.1%) due to the high resolution of the VNA.

Figure 2 also shows the “corrected” analytical calculations of the resonant frequency by modifying the expression for strip line fringing capacitance *C*_*f*_ in Equation 5 to an empirical^35^:

11 $${C}_{f}={\varepsilon }_{0}\frac{2\pi w}{\mathrm{log}(2.4w/h)},$$

which provides an improved fit to the data. Exploration of different expressions for the fringing are common in analyses of this type, depending on the *w*/*g* ratio, and other fringing capacitance corrections could be explored to improve the fit further^36^.

### Anisotropy

**Figure 3:**
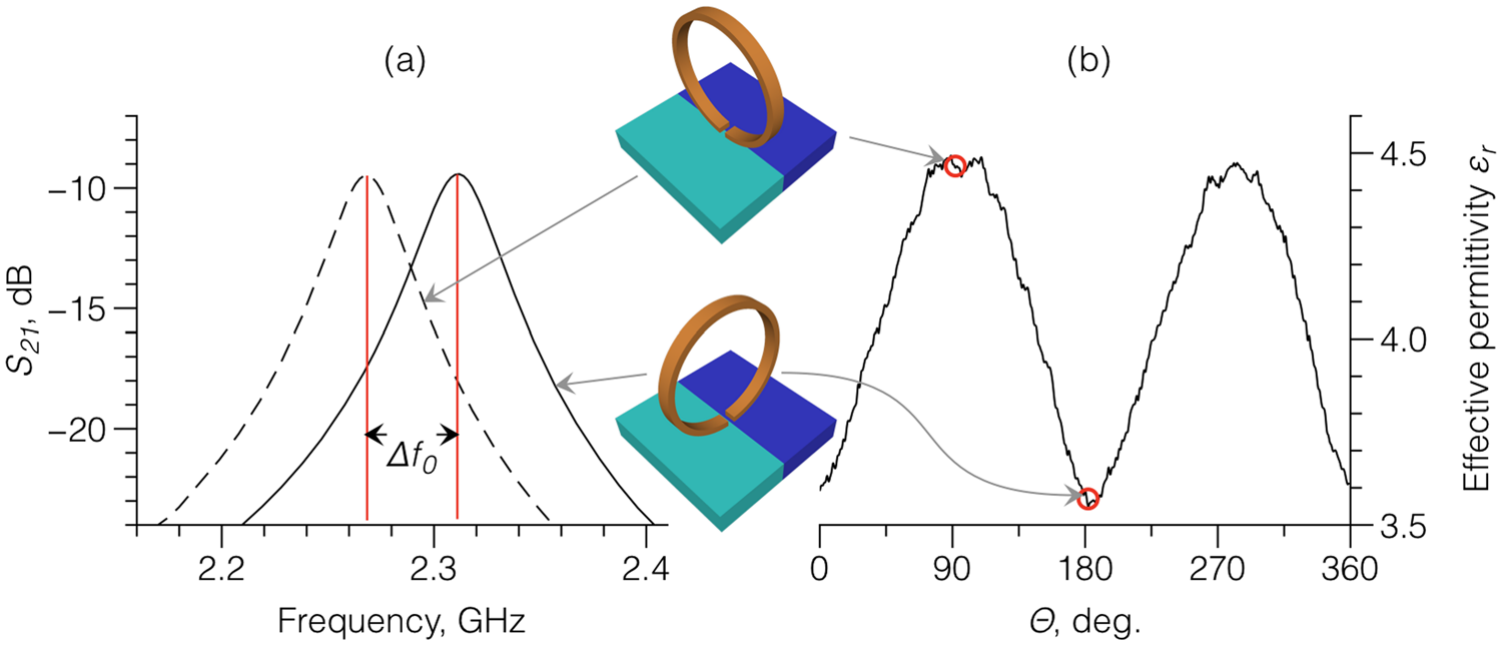
(**a**) Simulation of the transmission coefficient *S*_21_ for a SRR dielectric probe oriented parallel (left-shifted curve) and perpendicular (right-shifted curve) to the boundary between two materials with different permittivities *ε*_1_ = 2.6 and *ε*_2_ = 7.2; and (**b**) the measured effective relative dielectric permittivity at the interface region of the two materials *ε*_1_ and *ε*_2_ as a function of the rotation angle.

The projection of the SRR slit lying flat on the *xy* surface plane of the sample is described by a rectangle *w* × *g*. The electric field excited in the SRR gap is largely oriented perpendicular to the gap walls (as shown in Fig. 1b) and thus the SRR probe should be sensitive to the anisotropy of the dielectric susceptibility of the sample. This is confirmed by simulation and experiment shown in Fig. 3, which considers the gap sitting on the interface between two materials (blue and green) with distinctly different relative dielectric permittivities *ε*_1_ = 2.6 and *ε*_2_ = 7.2 respectively (typical values of permittivity for 3D-printed dielectrics^6^) with the electric field between the faces of the gap initially perpendicular to interface between the two materials. Figure 3a shows the simulated change for SRR 4 in the transmission signal *S*_21_ as the ring is then rotated through 90°. The rotation caused a shift of the resonant frequency Δ*f*_0_ = 40.5 MHz, which corresponds to a difference in dielectric permittivity of Δ*ε* = 0.885.

Figure 3b shows the measured effective relative dielectric permittivity of the interface region as the gap is rotated progressively, directly on the interface, through 360°. The permittivity had a pronounced polarization dependence on the orientation of the gap (i.e. the electric field), with a measured value of Δ*ε* = 0.9 in excellent agreement with simulation in Fig. 3a, and suggesting that the SRR technique could also be used as a probe for dielectric surface anisotropy.

**Figure 4:**
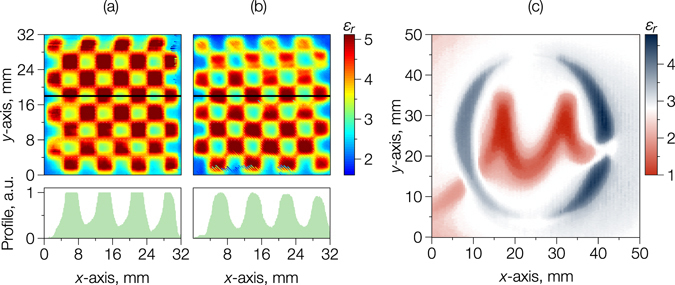
The spatial distribution of the relative dielectric permittivity in a 3D-printed chessboard with alternating cells of *ε*_1_ = 2.6 and *ε*_2_ = 5.2, using an SRR probe with *w* × *g* = 1.9 × 0.3 mm^2^ gap oriented (**a**) normal to the vertical cell boundaries and (**b**) at 45° to the cell boundaries (the bottom insets show the profiles along the black horizontal lines in the maps); (**c**) the relative dielectric permittivity map of a 3D-printed logotype of the Oxford Materials composed of “three-colour” materials with *ε*_1_ = 2.6, *ε*_2_ = 5.0 and *ε*_3_ = 1.

In general, the elongated gap geometry and the resulting strong orientation of the coupled electric field in the SRR gap must be taken into account when surface relative dielectric permittivity experiments are performed. Figure 4 shows 2D maps of measured relative dielectric permittivity over a 3D-printed 32 × 32 mm^2^ area “chessboard” surface composed of cells of either *ε*_1_ = 2.6 or *ε*_2_ = 5.2. The scan step was 0.2 mm and the map took approximately 20 minutes to acquire. The data in Fig. 4a,b were obtained with the SRR gap oriented normal to the horizontal call boundaries and at 45° to the cell boundaries respectively. Qualitatively, it was immediately apparent that at the 45° orientation in Fig. 4b, a relatively diffuse interface between cells was resolved, which is emphasised in the profile line taken across the superimposed horizontal black lines on the chessboard. Quantitatively and as previously demonstrated, the resolved relative dielectric permittivities were in good agreement with those obtained by standard bulk techniques, even accounting for differences in the measurement frequency (because the relative permittivity had a low sensitivity to frequency in the range studied).

Figure 4c shows an image of a surface with high contrast in dielectric permittivity where the Greek letter ‘*μ*’ is air (*ε*_*r*_ = 1) and the ‘O’ is a ABS/BaTiO_3_ composite (with *ε*_*r*_ = 5.02) framed by ABS polymer (*ε*_*r*_ = 2.65), representing the logo of the Department of Materials at Oxford University with an overall area of 50 × 50 mm^2^ and measured using SRR 1 (see Table 2) with a 0.25 mm step resolution.

### Restrictions and further improvements

**Figure 5:**
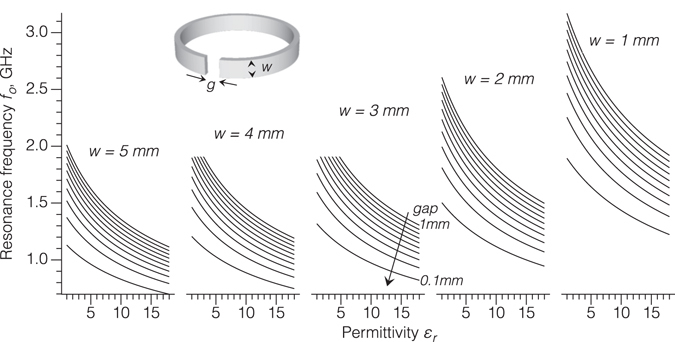
Calculated dependence of the resonant frequency of the SRR probe with different values of ring width *w* and slit gap *g* on dielectric permittivity of the material sample.

The simple SRR probe design allows easy modification of the probe geometry, for example, to provide a high sensitivity to the sample permittivity with fine spatial resolution, or to map a large-scale dielectric surface quickly with a coarser scanning step. As seen from Equations (1) to (4), by varying *r*, *h*, *w*, and *g*, it is possible to choose the operating frequency, increase the Q-factor or expand the *f*_0_(*ε*_*r*_) dispersion. This flexibility is demonstrated further in the simulations in Fig. 5 where *f*_0_(*ε*_*r*_) is shown for different combinations of *w* × *g* at constant *r* = 10 mm and *h* = 0.8 mm. A narrow gap with *w* × *g* = 5 × 0.1 mm^2^ provided a narrow band Δ*f*_0_ ≈ 0.4 GHz and a decrease in operating frequency to approximately 1 GHz, while at *w* × *g* = 1 × 1 mm^2^ the SRR probe blue-shifted the operational frequency and broadens Δ*f*_0_ to 1.2 GHz. As was also supported by experiment, a decrease in *w* × *g* from 5 × 0.1 to 1 × 1 mm^2^ resulted in a decrease in Q-factor from 100 to 20. This is in accordance with Equation (3) and that the conductive loss of the unloaded SRR (*Q*_un_) is proportional to the inductance: *Q*_un_ = 2*πf* · *L*/*R*, where *R* is a ring resistance. Similarly, it is expected that the Q-factor will decrease with decreasing gap width and height, and will increase with the SRR radius. Thus, the operating frequency and Q-factor of the SRR probe can be easily tuned simply by changing the SRR geometry.

**Figure 6:**
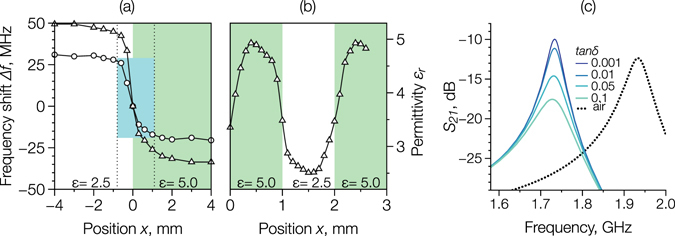
Simulated resonant frequency shift Δ*f*_0_ in a material composed of alternating strips with *ε*_1_ = 2.5 and *ε*_2_ = 5.0, for SRR probes with *w* = 5 mm, *h* = 0.8 mm, *g* = 0.35 mm, and radius *r* = 7 mm (triangles) and *r* = 10 mm (circles) where the width is (**a**) 4 mm and (**b**) 1 mm; (**c**) simulated transmission parameters *S*_21_ near the resonance frequency of the SRR probe in contact with a material with *ε*_*r*_ = 2.5 and tan *δ* in the range 0.1 to 0.001.

As shown earlier, the spatial resolution of the SRR probe will be determined by the gap area and its relative orientation to any sharp interface between different permittivity materials, and will affect the apparent interface or boundary “width”, even if this boundary is sharp. Figure 6(a) shows numerical simulations of the resonant frequency shift Δ*f*_0_ for a boundary structure with *ε*_1_ = 2.5 and *ε*_2_ = 5.0, for two SRR probes with *w* = 5 mm, *h* = 0.8 mm, *g* = 0.35 mm, and a radius *r* = 10 or 7 mm, scanning along the *x*-direction (with the gap perpendicular to the boundary). Figure 6(a) shows the apparent “width” of the interface (light blue) was suggested as 2 mm for both SRR probes. Figure 6(b) similarly shows simulations of the effective permittivity profile for a structure again with alternating regions of *ε*_1_ = 2.5 and *ε*_2_ = 5.0 for the scan orientation along the x-direction but where the width of each domain is reduced from 4 mm to 1 mm. Now, the profile *ε*_*r*_(*x*) did not demonstrate any region where the apparent permittivity was constant and the interface was smeared. These numerical results are in good agreement with experiment in Fig. 4 and for previous related demonstrations^6, 37, 38^. Typically, the regions close to a boundary with high permittivity contrast (e.g. the edge of the sample, where the low-permittivity region is air) show lower effective permittivity (see, for example, the small areas enclosed by the internal edges of the chessboard and the letter ‘*μ*’ in the logo in Fig. 4). This smearing effect can be minimised to some extent by selection of the SRR geometry.

The SRR probe can also be used for an evaluation of the dielectric loss in a material. Figure 6(c) shows the simulated resonance peak of the transmission signal *S*_21_ for a SRR with *r* = 1 mm, *w* = 3 mm, *h* = 0.8 mm, and *g* = 0.3 mm, for the probe in free space (dotted line, *f*_0_ = 1.933 GHz) and in contact with the material with a real part of dielectric permittivity *ε*′ = 2.5, for different values of the dielectric loss expressed by $\tan \,\delta =\varepsilon ^{\prime\prime} /\varepsilon ^{\prime}$, where *ε*′ and *ε*′′ are the real and imaginary parts of the relative dielectric permittivity. The total loss of the SRR is represented by a quality factor *Q* that fulfils:

12 $${Q}^{-1}={Q}_{c}^{-1}+{Q}_{d}^{-1}+{Q}_{r}^{-1},$$

where *Q*_*c*_ is the quality factor due to the conduction losses, ${Q}_{d}=1/\tan \,\delta$ is the quality factor due to the dielectric losses, and *Q*_*r*_ is the quality factor quality factor due to radiation losses of the resonator.

In free space, the resonator has a Q-factor of 35.6 at 1.933 GHz, but when in contact with a comparatively low loss dielectric material (*ε*′ = 2.5, $\tan \,\delta ={10}^{-3}$) its Q-factor surprisingly increases to 54.1 which would suggest that dielectric losses are playing a more important role in the overall SRR probe quality than might have been expected. Potentially, this could arise because the SRR gap itself behaves like an electric dipole antenna and it is a more efficient radiator when loaded with a dielectric material (that is lossy itself). To some extent, an increase of the Q-factor when in contact with a material, is a result of the lower resonance frequency in this condition (1.731 GHz) producing a lower surface resistance (which is proportional to $\sqrt{\omega }$) and reducing radiation efficiency for the SRR, which acts in this case as a simple loop antenna. For these two frequencies, the radiation resistance of simple loop antennas the same size as the SRR would be *R*_*r*_ = 3.97 Ω (1.731 GHz) and *R*_*r*_ = 5.9 Ω (1.933 GHz), equivalent to a 32% reduction in radiation losses *Q*_*r*_. Additionally, a shift of the resonance to lower frequency causes an increase in the current flow skin depth and therefore promotes a more uniform current distribution inside the SRR, reducing the resistive loss *Q*_*c*_^39^. When a material with a comparatively high loss is introduced, there is, as expected, a decrease in the total Q-factor, following an inverse relationship and with the lowest Q-factor of 19.8 for a loss of tan *δ* = 0.1. Nonetheless, some uncertainty remains in the correct physical interpretation of the Q-factor data, and is the subject of continuing investigation.

## Conclusion

A simple measurement approach and supporting analytical expressions to image the surface electromagnetic properties of materials based on a single split-ring magnetic resonator have been proposed. Analytical calculations, numerical simulations and experimental measurements have been used to verify and explore the capabilities and limitations of the approach for surface relative dielectric permittivity mapping. The SRR probe was experimentally convenient and showed approximately 7.7% average accuracy in permittivity measurements in this preliminary implementation; permittivities of approximately 2 to 27 were measured in the frequency range 1–3 GHz. The spatial resolution of the technique was in the range of 0.1–2 mm and was tunable depending on the geometric parameters of the SRR itself. The SRR dielectric probe approach was suggested to be particularly well-suited to applications requiring 2D near-field dielectric imaging of large area composite materials.

## Methods

The SRR elements that form the key part of the dielectric probe were simply obtained as transverse sections of commercial purity Cu pipe using a low speed circular saw (IsoMet, Buehler). A range of diameters and wall thicknesses were investigated, and in each case the radius, height and width of the SRR were measured using laboratory callipers to a precision of ±0.01 mm. Once the rings were cut axially to generate the air gap (or slit), the gap was measured using an optical microscope to approximately 1 *μ*m accuracy.

The SRR element was mounted in specially designed and 3D-printed polymer holder, between two 3 mm diameter magnetic loops (coils) connected to a Rohde&Schwarz ZNB20 vector network analyser (VNA). The *xy*-plane scanning of this probe assembly was accomplished using NEMA17 stepper motors operated through a programmable motion controller. For the mapping of the surface dielectric properties of materials, the resonant frequency of the complex insertion loss (transmission coefficient *S*_21_) as the resonating probe approached and touched the surface was measured point-by-point over the surface by automated synchronisation of the VNA with the motion controller. The overall scanning speed, typically determined by the response of the VNA and scanning step distance, was about 200 *μ*s per step.

Data was collected over the surface of composite materials composed of a matrix of acrylonitrile butadiene styrene (ABS) polymer filled with a dispersion of various high-dielectric fine-powdered ceramics such as BaTiO_3_ or CaTiO_3_, with different volume ratios to obtain a range of surface dielectric permittivities, according to our previous work^4–6^. To explore the accuracy of the technique, the dielectric permittivities of each of these different composites were characterised separately by the split-post dielectric resonance method^40^ at an operating frequency of 15 GHz. To explore the spatial resolution of the technique, the same composite materials were also arranged in various 2D patterns using a fused deposition modelling (FDM) based 3D printing technique, details of which we have published elsewhere^5, 6^.

A finite element method (FEM) based model to simulate the 3D distribution of the electric and magnetic fields in and around the SRR probe in contact with various surfaces of different dielectric permittivity was developed. Full wave simulations were performed using commercially available Comsol Multiphysics RF module software. The geometry of the probe arrangement used in simulation was derived from a 3D CAD model, while the FE mesh element size was carefully optimised to provide mesh-independent solutions. The outputs of the simulation were the complex transmission and reflection parameters from which a resonant frequency was retrieved, and was then used to help interpret and validate the experimental measurements.
